# Severe reversible cardiomyopathy associated with adrenal crisis caused by isolated adrenocorticotropin deficiency: a case report

**DOI:** 10.3389/fcvm.2025.1451635

**Published:** 2025-01-28

**Authors:** Li Wang, Fangfang Bu, Lanjie He, Guihua Yao

**Affiliations:** ^1^ Department of Cardiology, Qilu Hospital (Qingdao), Shandong University, Qingdao, China; ^2^ Department of Endocrinology, Qilu Hospital (Qingdao), Shandong University, Qingdao, China; ^3^ The Key Laboratory of Cardiovascular Remodeling and Function Research, Jinan, China

**Keywords:** adrenal crisis, acute adrenocortical insufficiency, reversible cardiomyopathy, heart failure, isolated acth deficiency

## Abstract

Adrenal crisis, also known as acute adrenal insufficiency, is an endocrine emergency that is associated with high mortality rates. Reversible cardiomyopathy with severe heart failure is a rare complication of adrenal crisis. Isolated adrenocorticotropin deficiency (IAD) is a rare condition of pituitary adrenal insufficiency. In this case report, we describe a 74-year-old male patient who was in good physical health and was admitted to our hospital with a sudden onset of fever and confusion that was complicated by hyponatremia and hypotension. Cardiac ultrasound showed significantly reduced left ventricular ejection fraction (LVEF; 10%). The patients was initially diagnosed with “septic shock” because of elevated inflammatory indicators and treated with mechanical circulatory support, antibiotics, fluid resuscitation, and intravenous administration of 50 mg hydrocortisone every 6 h for 2 days (400 mg in total). The symptoms of the patient improved significantly by this treatment in 6 days. The LVEF improved from 10% to 40%. However, the initial treatment did not alleviate hypotension and confusion. Therefore, the status of adrenal function was analyzed using blood and urine cortisol tests. Blood and urinary cortisol levels were significantly reduced, but concurrent increase in the ACTH levels were not observed. This indicated adrenal crisis. Subsequently, the patient was initially administered intravenous injection of hydrocortisone (50–150 mg/day) for 5 days, and then transitioned to a physiological supplement dose orally. The LVEF value improved further to 52%. Finally, the patient was diagnosed with adult isolated ACTH deficiency. The patient was prescribed regular oral hydrocortisone. The patient has not shown any signs of heart failure during follow up for more than half a year. In summary, we described a rare and severe case of adrenal crisis complicated with reversible cardiomyopathy that was caused by isolated ACTH deficiency. In such a case, conventional guideline directed medical therapy (GDMT) for heart failure was not considered suitable because of the underlying hypotension, hypoglycemia, and hyponatremia. Our study showed that timely supplementation of glucocorticoids achieved better therapeutic effects in patients with adrenal crises complicated by severe cardiomyopathy.

## 1 Introduction

Adrenal insufficiency is a disease in which the adrenal glands do not produce optimal levels of adrenal hormones such as cortisol because of damage to the adrenal glands (primary cause), pituitary gland dysfunction (secondary cause), or inhibition of adrenal gland function by the exogenous glucocorticoids (tertiary cause) (1, 2). Isolated adrenocorticotropic hormone (ACTH) deficiency (IAD) is a rare type of secondary adrenocortical insufficiency that is characterized by low ACTH and cortisol levels accompanied by normal secretion of other pituitary hormones (3). When compared with other types of adrenal insufficiency, the symptoms of IAD are relatively mild in the adults. The onset of IAD is insidious and the symptoms are either not detected or misdiagnosed. Adrenal crisis is the most severe manifestation of adrenal insufficiency and is often associated with high mortality rates. In rare cases, adrenal crisis is the primary manifestation of IAD. Cardiovascular manifestations of adrenal crisis are hypotension and arrhythmias, but cases of severe cardiomyopathy are rare and reported in only a few studies (4). In this case report, we describe a rare case of severe cardiomyopathy that was secondary to the IAD associated adrenal crisis.

## 2 Case description

A 74-year-old man in good health except for chronic atrial fibrillation was admitted to our hospital with a sudden onset of fever and temporary loss of consciousness. He developed a fever on June 15, 2022, which progressed to nausea, vomiting, confusion, and inability to speak by the next day. He was rushed to a local hospital where his blood glucose level was 3.1 mmol/L and blood pressure was 80/50 mmHg. The local hospital provided dopamine for increasing blood pressure and ampicillin for anti-infection treatment. However, the effects were not favorable. Therefore, on June 17, 2022, he was transferred to the intensive care unit (ICU) of our hospital. His vital signs on admission were as follows: blood pressure (BP), 75/50 mmHg (maintained by vasopressor); temperature, 37.5 ℃; heart rate (HR), 120 beats/minute; and respiratory rate (RR), 28 breaths/minute. Physical examination at admission showed the following clinical characteristics: BMI, 17.3 kg/m^2^; lethargic; moist rale in both lungs; irregular heartbeat; absence of edema in the lower limbs; cold extremities; presence of a small number of visible piebald spots; absence of neck stiffness; negative pathological signs.

A complete blood count showed reduced lymphocyte counts (0.78 × 10^9^/L) but the counts of white blood cells and neutrophils were normal. Furthermore, we observed elevated levels of C-reactive protein (CRP; 53.9 mg/L, normal range <8 mg/L), procalcitonin (PCT; 3.39 ng/ml, normal range <0.1 ng/ml), brain natriuretic peptide (BNP; 803 pg/ml, normal range < 100 pg/ml), and myoglobin (>500 ng/ml, normal range < 107 pg/ml), but the levels of creatine kinase isoenzyme (CK-MB) and high-sensitivity troponin I (hs-cTnI) were within the normal range. Biochemistry tests demonstrated low serum levels of sodium (122 mmol/L, normal range135–155 mmol/L), high levels of total bilirubin (44 μmol/L, normal range 5–21 μmol/L), and normal levels of potassium, glucose, urea nitrogen and creatinine. D-Dimer levels were within the normal range. The patient underwent two sets of blood cultures (bilateral, anaerobic and aerobic), three urine cultures, and two sputum cultures; however, no positive results were identified. Additionally, we conducted G tests, GM tests, respiratory pathogen serology, nucleic acid testing, and next-generation sequencing (NGS), yet no significant pathogens were detected.

**Figure 1:**
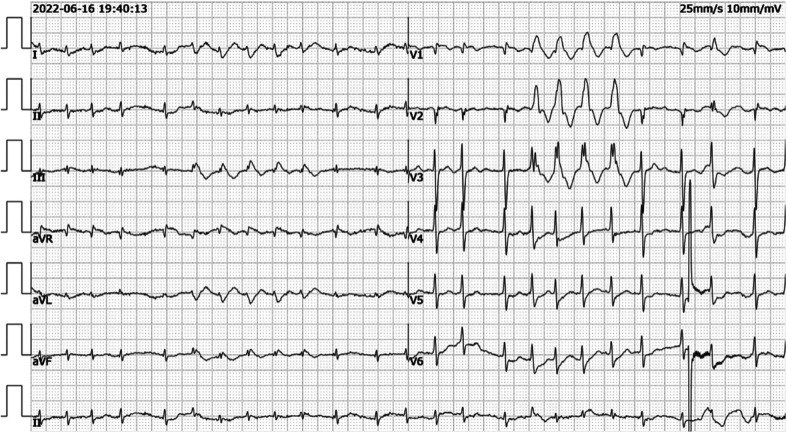
Electrocardiogram showed tachycardic atrial fibrillation with differential conduction a few times, mild changes in ST-T, abnormal Q waves in leads V1-V2, and incomplete right bundle branch block.

Electrocardiogram showed tachycardic atrial fibrillation with differential conduction a few times, mild changes in ST-T, abnormal Q waves in leads V1-V2, and incomplete right bundle branch block (Figure 1).

**Figure 2:**
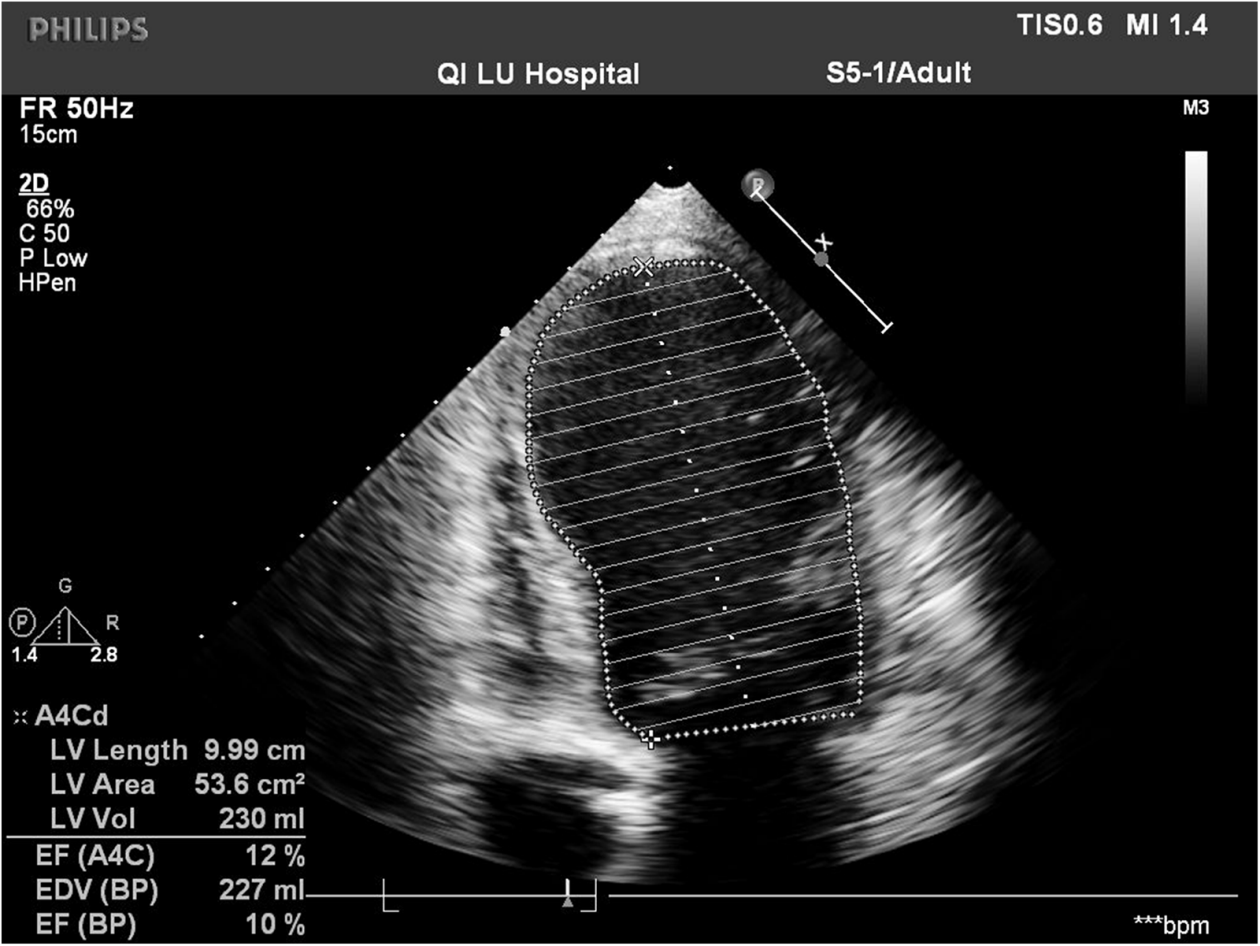
Bedside cardiac ultrasound showed significantly low left ventricular ejection function (LVEF 10%).

Chest CT scan showed pulmonary edema or pulmonary infection, a significant increase in the cardiothoracic ratio, and bilateral pleural effusion. CT scan of the head, abdomen, and pelvis did not show any significant abnormalities. Bedside cardiac ultrasound showed significantly low left ventricular ejection function (LVEF;10%) (Figure 2), slightly enlarged left ventricular end-diastolic anteroposterior diameter (LVEDd; 53 mm), and reduced diffuse wall motion in the left and right ventricles.

Based on the clinical history and medical examination results described above, the patient was diagnosed with septic shock combined with unexplained heart failure. Since the pathogen of infection was unknown, the patient was administered full coverage and de-escalation treatment, including meropenem, vancomycin, and peramivir. He was also administered adequate fluid resuscitation with “cocktail therapy,” including an intravenous injection of 400 mg hydrocortisone every 6 h for two days. The patient was diagnosed with severe heart failure, metabolic acidosis, and respiratory failure. Therefore, we performed hemofiltration, mechanical ventilation, and intra-aortic balloon pump (IABP), and administered large doses of vasoactive drugs to maintain the blood pressure. During the course of the disease, the patient developed acute liver injury, thrombocytopenia, and coagulation dysfunction. Therefore, he was treated with liver protection treatment (reduced glutathione), platelet transfusion, and the coagulation factor supplement.

After careful treatment for six days, the symptoms of the patient improved significantly. NT-proBNP levels decreased from 26,400 pg/ml at the peak to 5,520 pg/ml. The LVEF value improved to 40%. The left ventricular diameter (LVEDd) normalized to 48 mm. The chest CT results were normal. The body temperature and several inflammatory indicators, including CRP and PCT, returned to normal.

However, the treatment did not resolve problems such as low blood pressure, confusion, speech loss, and the inability to eat and move. Furthermore, the cause of heart failure was still unknown. Therefore, to resolve these health problems, the patient was transferred to the Department of Cardiology at our hospital. The medical history of the patient was reviewed again, including the onset of acute infection and symptoms such as hypotension, hypoglycemia, hyponatremia, nausea, and vomiting. The patient was usually silent, disinterested in activity, and showed low blood pressure and poor appetite. Physical examination showed emaciation, and sparse pubic and axillary hair. Therefore, we performed the blood and urine cortisol tests to determine if the adrenocortical function was suppressed in the patient. The blood cortisol level at 8 am was significantly low (1.7 μg/dl; normal range: 4.26–24.85 μg/dl). The 24-h urinary free cortisol level was also low (2.9 μg/24 h; normal range: 3.5–54.0 μg/24 h). Blood ACTH level was in the normal range but closer to the lower limit (13.6 pg/ml; normal range: 7.2–63.4 pg/ml). Therefore, the patient was diagnosed with acute adrenocortical insufficiency. However, it was not yet clear if this condition was primary or secondary. Therefore, we performed follow-up imaging tests. Adrenal CT scan did not show any significant abnormalities. The pituitary MRI did not show any pituitary mass, hemorrhage, empty sella, or other pathological conditions. Coronary angiography did not show significant stenosis. Furthermore, other pituitary gland-related hormones from the gonadal axis and the growth hormone axis were within the normal range. However, hormones related with the thyroid axis showed significant changes, including increased TSH (80.8 μIU/ml), decreased T3 (4.34 pmol/L) and T4 (6.66 pmol/L), and positive thyroglobulin antibody (TGAb) and thyroid peroxidase antibody (TPOAb). Therefore, the patient was diagnosed with secondary adrenocortical hypofunction and potential isolated ACTH deficiency.

**Figure 3:**
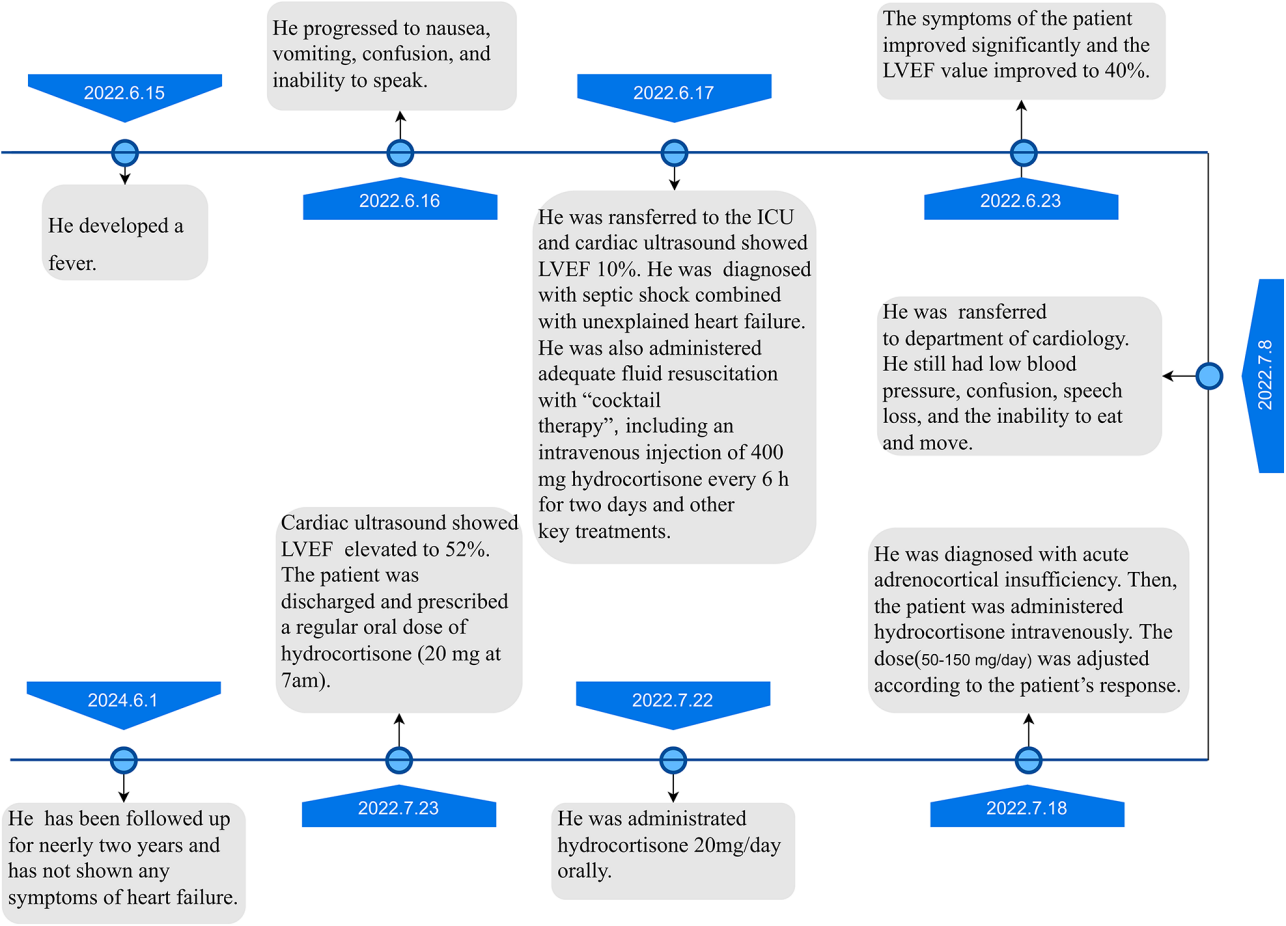
Timeline.

Then, the patient was administered hydrocortisone intravenously. The hydrocortisone dose (50–150 mg/d) was adjusted according to the patient's response. Subsequently, the patient was transitioned to a physiological supplement of hydrocortisone through the oral route. The patient's speech and muscle strength recovered quickly. He started walking independently on the 5th day after hydrocortisone administration. The serum sodium level and blood pressure returned to the normal range without extra sodium supplement and vasopressors. The LVEF value recovered significantly to 52%. The ventricular wall activity also returned to normal. The cortisol hormone level decreased in the patient but ACTH concentration did not increase as expected from the feedback mechanism. Therefore, after reviewing the literature, the patient was diagnosed with isolated ACTH deficiency. The patient was prescribed a regular oral dose of hydrocortisone (20 mg at 7am). The patient has been followed up for neerly two years and has not shown any symptoms of heart failure. The timeline is attached at the end (Figure 3).

## 3 Discussion

Adrenal crisis, also known as acute adrenocortical insufficiency, is an acute physiological disorder caused by the lack of cortisol, an adrenal hormone. It is an endocrine emergency that is associated with high mortality rates and requires immediate diagnosis and treatment. Adrenal crisis is characterized by fatigue, weakness, gastrointestinal symptoms (nausea, vomiting, abdominal pain), hypotension, hypoglycemia, hyponatremia, hyperkalemia, disturbance of consciousness, shock, and other physiological symptoms. It is often misdiagnosed or not detected clinically because of non-specific symptoms. The guidelines for management of patients with hyponatremia includes screening the levels of cortisol (2).

In addition, sometimes cortisol hormone is not absolutely deficient (below the lower limit of normal), but relatively insufficient (within the normal range), which often happens in some critically ill patients. In 2008, the American Society of critical Care Medicine proposed a new concept of critical illness related corticosteroid insufficiency (CIRCI) (5). In 2017, the American Society of Critical Care Medicine/European Society of Critical Care Medicine updated the CIRCI guidelines and defined CIRCI as a relative failure to meet the demand rather than an absolute decrease in the cortisol levels (6). However, the guideline emphasizes that CIRCI denotes a relative inability to meet the demand for cortisol rather than an absolute reduction in circulating cortisol levels. However, The guideline points out that CIRCI refers to a relative failure to meet the demand rather than an absolute decrease in the level of cortisol in the body. The diagnostic criteria, timing of initiation of treatment, steroid dosage, and course of treatment for these patients are still controversial. Several studies have shown that “cocktail therapy” with a regimen of hydrocortisone, vitamin C, and vitamin B1 helps alleviate sepsis shock (7). In this case study, the patient was initially diagnosed with sepsis but did not respond well to vasoactive drugs. He was also treated for 2 days with “cocktail therapy,” which was discontinued after his blood pressure improved. However, at that time, hydrocortisone treatment was only used to improve the shock state but regular oral hydrocortisone treatment was not initiated. Therefore, in retrospect, we missed the opportunity for etiological diagnosis. But, the early administration of hydrocortisone improved the left ventricular ejection fraction from 10% to 40%.

The spectrum of clinical symptoms for adrenal insufficiency is broad. In adults, cardiovascular manifestations of adrenal insufficiency include arrhythmia, hypotension, or syncope, but cases of severe cardiomyopathy are rare. Cardiovascular manifestations are also reported in some cases of children with adrenal insufficiency (8–11). However, adrenal insufficiency in children is mostly caused by congenital defects, and their clinical manifestations and outcomes are different from those of adults. We have not included these details in this paper. Including our case, we identified eleven reports of adult isolated ACTH deficiency accompanied with severe cardiomyopathy in the literature (see Table 1). We reviewed the literature and found that cardiomyopathy mainly appeared at two time points: first, about 2 weeks after the initiation of fludrocortisone treatment (21); The second is secondary to adrenal crisis. For the first condition, severe heart failure is mainly considered to be due to the side effects such as strong water and sodium retention of mineralocorticoid hormone, which is not the same as the pathogenesis of this case. This paper does not summarize such cases in detail. A total of 10 cases of severe cardiomyopathy secondary to adult adrenal crisis were retrieved through systematic literature search, and the pathophysiological mechanism, clinical manifestations, treatment strategies and prognosis of this disease were summarized.

**Table 1:** Summary of previous and current cases of adrenal crises with cardiomyopathy.

No.	Reference	Report year	Age/sex	Cause	Manifestations	Electrocardiogram	Echocardiogram or Left ventricular angiography	Treatment	Outcome
1	Cushner et al. (12)	1963	53/M	Tuberculosis of adrenal gland	Severe cough, productive of white frothy sputum, and with dyspnea, palpitations, orthopnea, nocturnal dyspnea, fever.	Supraventricular tachycardia with a rate of 170 per minute and ST-T wave abnormalities.	N/A, But chest x-ray showed marked cardiac enlargement, pulmonary edema, and right pleural effusion.	(1) diuretic, anticoagulation therapy, quinidine, digitalis (2)9-Fluorohydrocortisone, 0.2 mg 3 times.	7 days after receiving 9-FLudrocortisone, he died suddenly.
2	Iga et al. (13)	1992	74/F	IAD	Low grade fever, general fatigue and loss of appetite, no significant heart-related symptoms.	1. Deep negative T waves in left precordial leads. 2. Frequent premature ventricular contractions with long QT intervals	Takotsubo cardiomyopathy	Steroid hormone supplement (no details)	5 days, Left ventricular motion returned to normal, and 4 weeks, the deep negative T waves normalized.
3	Iga et al. (13)	1992	64/F	IAD	Loss of consciousness due to hypoglycemia.	Diffuse ST segment elevation in left precordial leads	Takotsubo cardiomyopathy	Steroid hormone supplement (no details)	2 weeks later, Left ventricular wall motion abnormalities normalized
4	Tokushi Koga et al. (14)	2000	62/M	Empty Sella	Congestive heart failure with lung edema	Prolonged QTc interval (0.62 s) and negative T wave on the right precordial leads, frequent multifocal ventricular premature contractions (1,7000 beats/day).	LVEF = 37% and LVDd = 60 mm	Hydrocortisone 20 mg/day	2 months later, left ventricular size was reduced to normal range (LVDd 51 mm) and systolic function was improved (LVEF 52%)
5	Sakihara et al. (15)	2007	53/F	Empty Sella	Loss of consciousness due to hypoglycemia	ST segment elevation and T wave inversion in leads V1-6	Takotsubo cardiomyopathy	Hydrocortisone 200 mg/day intravenously for one week and 15 mg/day per os.	2 weeks later, LV wall motion recovered, and LVEF improved to 70%
6	Ukita et al. (16)	2009	69/F	IAD	General fatigue, loss of appetite, chest pain.	T waves were inverted in leads V1 and 2, and diphasic in leads V3-4. Ventricular tachycardia	Takotsubo cardiomyopathy, and LVEF = 33%	Intravenous hydrocortisone 100 mg four times daily. then 100 mg three times daily. hydrocortisone 20 mg/day per os	8 days later, LVEF restored to 74% 3 weeks later, left ventricular wall motion returned to normal.
7	Laway et al. (17)	2010	25/F	Sheehan syndrome	Fatigue, weakness, unconscious	Heart rate of 90 per min with low voltage complexes.	Dilated cardiomyopathy with LVEF = 23% and LV = 59	Levothyroxine100 mg/day, Prednisolone 7.5 mg/day	4 months later, LVEF 41%. 7 months later, LVEF 65%.
8	Shimizu et al. (18)	2011	54/F	IAD	Consciousness disturbance, fever, hypoglycemia, hyponatremia, rhabdomyolysis, hypotension	Inverted T waves in leads II, III, and aVF	Dilated cardiomyopathy FS = 12%, right-sided atrial and ventricular dilatation.	Initiated with 100 mg of intravenous hydrocortisone every 8 h changed to oral hydrocortisone 15 mg/day.	8 days later, global myocardial dysfunction completely returned to normal.
9	Krishnamoorthy et al. (19)	2013	21/M	Addison's Disease	Nausea, weakness, progressive dyspnea, asystolic cardiac arrest, pericardial effusion, severe biventricular failure, and cardiogenic shock.	N/A	Biventricular dilation, pericardial effusion, and increased ventricular wall thickness.	(1) TandemHeart implantation (2) IV hydrocortisone loading dose 100 mg and there after 50 mg/8 h. (3) Weaned to physiologic dose of oral hydrocortisone by discharge.	(i) At 2 weeks after RVAD and LVAD removal, normal biventricular function (ii) At 2 months, remained well.
10	Wang et al. (20)	2021	68/F	Tertiary adrenal insufficiency	Frequent heart failure and shock.	Complete left bundle branch block.	Dilated cardiomyopathy with LVDd = 68 mm and LVEF = 33%.	Spironolactone (20 mg once daily), hydrocortisone (50 mg) was administered intravenously every 6 h, and other vasoactive drugs.	18 months later, LVEF 35%, LVDd 64 mm. However, died of sudden cardiac death.
11	Present case	2024	74/M	IAD	Fever and disturbance of consciousness	Atrial fibrillation with tachycardia	Regional ventricular wall motion abnormality, and LVDd = 53 mm, and LVEF = 10%.	Hydrocortisone 50–150 mg/day, the dose was adjusted according to the patient's response, then changed to oral hydrocortisone 20 mg/day.	5 days later, the ventricular wall activity generally returned to normal with LVEF 52%.

There are many hypotheses to explain the pathophysiological mechanisms of cardiomyopathy that is secondary to the adrenal crisis. The role of glucocorticoids on the cardiac function are summarized as follows: (1) Glucocorticoids act directly on the left ventricular myocardium and regulate its function (22, 23). Sarcoplasmic reticulum (SR) is the main target of glucocorticoids in the heart. Glucocorticoids regulate SR-Ca^2+^ uptake and recycling, and myocardial contraction by modifying the CaM kinase II system in the SR; reduced uptake of Ca^2+^ by SR decreased myocardial contractility (2) Glucocorticoids enhance the positive inotropic effects of catecholamines on the heart, and vice versa. (3) Mouse model studies showed that knockout of the myocardial glucocorticoid receptor caused myocardial hypertrophy, fibrosis, and heart failure, thereby demonstrating the protective effects of glucocorticoids on the myocardium. Due to the extensive effects of glucocorticoids, the current basic research is still relatively plain and simple, and more clinical and animal experiments are needed for further exploration.

The clinical manifestations of cardiomyopathy secondary to adrenal crises are as follows: (1) Acute onset and severe condition: adrenal crisis is often manifested as hypovolemia and gets worse when combined with pump failure. Furthermore, adrenal crisis is often accompanied by multiple organ failure because of the extensive effects of the glucocorticoids. Therefore, these patients need urgent mechanical circulatory support and comprehensive management in the ICU. (2) Echocardiography showed dilated cardiomyopathy, Takotsubo cardiomyopathy, or regional ventricular wall motion abnormality with reduced ejection fraction (except in some cases where no LVEF was given).

The treatment of adrenal crisis with severe cardiomyopathy also has its characteristics: (1) Steroid hormone is the key treatment. Hydrocortisone is relatively safe in patients with such cardiomyopathy, However, fludrocortisone often causes significant water and sodium retention and may aggravate heart failure. According to clinical recommendation in the literature, an initial dose of intravenous 100 mg hydrocortisone is followed by a dose of 200 mg hydrocortisone that is administered as a continuous infusion every 24 h or administered as intravenous boluses of 50 mg every 6 h. If the initial treatment is successful (usually after 24 h), an oral hydrocortisone dose that is 2–3 times the usual dose is first administered and then tapered down to the usual dose over the next 2–3 days. However, it is necessary to pay attention to the retention of water and sodium and monitor for any mental excitement during the use of the drug and adjust the drug dose accordingly in a timely manner. (2) Conventional guideline-directed medical therapy (GDMT) for heart failure may not be suitable for adrenal crises with cardiomyopathy because it is characterized by pump failure and severe hypotension. Therefore, vasodilators and diuretics should be used with caution in the acute phase, and positive inotropic drugs such as digoxin may be appropriate. The tetragenous therapy for HFrEF recommended in the 2022 AHA/ACC/HFSA heart failure guidelines, includes angiotensin receptor-neprilysin inhibitor (ARNI), *β*-blockers, sodium-glucose cotransporter-2 inhibitors (SGLT2), mineralocorticoid receptor antagonist (MRA) and other drugs (24). However, this therapy may not be suitable for patients with adrenal insufficiency accompanied by severe cardiomyopathy because of hypotension, hypoglycemia, and mineralocorticoid hormone deficiency. For patients presenting with acute heart failure accompanied by hyponatremia, hypotensive shock, alterations in consciousness, and an inadequate response to conventional treatment, we need to be vigilant regarding adrenal crisis-related cardiomyopathy and perform the tests of ACTH and cortisol rhythms.

Adult isolated ACTH deficiency (IAD) is a secondary adrenal insufficiency disease. The epidemiology and etiology of IAD remains uncertain at present because of rare and isolated cases. In 2005, the prevalence of adult IAD was estimated to be 7.3 per 100,000 subjects (average 10 years) in Tokyo and 3.8 per 100,000 subjects in central Japan. The pathogenesis of the disease is not clear and may be related to autoimmunity (25), which is difficult to confirmed. Autopsy has suggested that it may be related to lymphocytic hypophysitis, often combined with chronic lymphocytic thyroiditis (3). So far, gene mutations related to the pathogenesis of adult IAD have not been reported.

Adult IAD mostly occurs in the middle-aged and elderly people, and its clinical manifestations are comparable to those of primary adrenal insufficiency. However, the symptoms are milder and may not be easily noticeable until the occurrence of adrenal crisis because of the predisposing factors. It is characterized by extreme fatigue, nausea and vomiting, severe hypotension and hypoglycemia, and can be life-threatening if not treated immediately (26). Furthermore, the clinical manifestations of adult IAD are very nonspecific and variable and may include weakness, anorexia, abdominal pain, emaciation, and a tendency towards hypoglycemia. The symptoms are comparable with those observed in subjects with hypogonadism, but the sex hormones are usually normal. Adult IAD is often accompanied by increased TSH levels (43.8%) and nearly 60% of patients with IAD are positive for the thyroid autoantibodies (25).

In conclusion, we described a patient in this case report with adrenal crisis complicated by reversible and severe cardiomyopathy because of isolated ACTH deficiency. The patient showed a low LVEF value of 10% but recovered significantly after treatment with hydrocortisone. This is the first reported case in the Chinese population for adrenal crisis complicated with reversible but severe cardiomyopathy, which due to isolated ACTH deficiency. We postulate that conventional guideline directed medical therapy (GDMT) for heart failure may not be suitable for such patients because of complications including hypotension, hypernatrium, and hypoglycemia. Our data shows that timely supplementation of glucocorticoids achieved better therapeutic effects.

## Data Availability

The raw data supporting the conclusions of this article will be made available by the authors, without undue reservation.
